# Cortisol Regulates Cerebral Mitochondrial Oxidative Phosphorylation and Morphology of the Brain in a Region-Specific Manner in the Ovine Fetus

**DOI:** 10.3390/biom12060768

**Published:** 2022-05-31

**Authors:** Katie L. Davies, Danielle J. Smith, Tatiana El-Bacha, Peter F. P. Wooding, Alison J. Forhead, Andrew J. Murray, Abigail L. Fowden, Emily J. Camm

**Affiliations:** 1 Department of Physiology, Development and Neuroscience, University of Cambridge, Cambridge CB2 3EG, UK; kld47@cam.ac.uk (K.L.D.); djs253@cam.ac.uk (D.J.S.); tatiana@nutricao.ufrj.br (T.E.-B.); fbpw2@cam.ac.uk (P.F.P.W.); ajf1005@cam.ac.uk (A.J.F.); ajm267@cam.ac.uk (A.J.M.); 2 Department of Biological and Medical Sciences, Oxford Brookes University, Oxford OX3 0BP, UK

**Keywords:** cortisol, fetus, mitochondria, brain

## Abstract

In adults, glucocorticoids are stress hormones that act, partly, through actions on mitochondrial oxidative phosphorylation (OXPHOS) to increase energy availability. Before birth, glucocorticoids are primarily maturational signals that prepare the fetus for new postnatal challenges. However, the role of the normal prepartum glucocorticoid rise in preparing mitochondria for the increased postnatal energy demands remains largely unknown. This study examined the effect of physiological increases in the fetal cortisol concentration on cerebral mitochondrial OXPHOS capacity near term (~130 days gestation, term ~145 days gestation). Fetal sheep were infused with saline or cortisol for 5 days at ~0.8 of gestation before the mitochondrial content, respiratory rates, abundance of the electron transfer system proteins and OXPHOS efficiency were measured in their cortex and cerebellum. Cerebral morphology was assessed by immunohistochemistry and stereology. Cortisol treatment increased the mitochondrial content, while decreasing Complex I-linked respiration in the cerebellum. There was no effect on the cortical mitochondrial OXPHOS capacity. Cortisol infusion had regional effects on cerebral morphology, with increased myelination in the cerebrum. The findings demonstrate the importance of cortisol in regulating the cerebral mitochondrial OXPHOS capacity prenatally and have implications for infants born preterm or after glucocorticoid overexposure due to pregnancy complications or clinical treatment.

## 1. Introduction

In adults, glucocorticoids play a pivotal role in the response to stressful challenges, acting on multiple organ systems to redirect energy resources to meet a real or anticipated demand [1]. In the fetus, glucocorticoids can act as stress signals and adapt growth and development to aid intrauterine survival during adverse conditions [2]. In most mammalian species studied to date, fetal glucocorticoid concentrations also rise naturally towards term, which switches the cell cycle from accretion to differentiation in a range of fetal tissues in preparation for their new postnatal functions [3]. Consequently, the antenatal administration of potent synthetic glucocorticoids is widely used in women threatened with preterm delivery to improve neonatal viability [4]. However, the early activation of the maturational switch by glucocorticoid overexposure before term can have life-long consequences for tissue function with implications for adult health. Certainly, fetal exposure to supraphysiological glucocorticoid concentrations has been shown to impair the normal development of the brain and a number of other tissues [5].

Perinatal tissue differentiation and new postnatal functions require extra energy in the form of ATP, which is produced mainly by oxidative phosphorylation (OXPHOS) in the mitochondria [6]. Consequently, oxygen consumption increases neonatally in the whole body and by several individual tissues, including the skeletal muscle, liver and brain [7]. Glucocorticoids are known to affect bioenergetic function in a range of adult tissues, in part through actions on the mitochondrial OXPHOS capacity [8]. However, the role of cortisol during late gestation on mitochondrial function in fetal tissues such as the brain remains largely unknown. This study, therefore, tested the hypothesis that the mitochondrial OXPHOS capacity of the brain is responsive to raising cortisol concentrations within the physiological range in fetal sheep near term.

## 2. Materials and Methods

### 2.1. Animal Experimental Procedures

All experimental procedures were carried out under the Animals (Scientific Procedures) Act 1986 Amendment Regulations 2012 (licence number: PC6CEFE59), after ethical approval by the Animal Welfare and Ethical Review Body of the University of Cambridge, UK.

Between 114 and 119 days of gestation (dGA, term ~145 dGA), 13 time-mated Welsh mountain ewes carrying singletons were anaesthetised (1.5–2.0% isoflurane in O_2_:N_2_O) after an overnight fast and catheters were inserted into the fetal and maternal femoral arteries and veins as described previously [9]. Following a recovery period of at least 5 days, fetuses were randomly assigned to receive a 5-day continuous infusion of cortisol (2–3 mg/kg/day Solu-Cortef; Pharmacia, Kent, UK) or saline (0.9% NaCl, 3 mL/day) into the femoral vein. All catheters were flushed daily, and blood samples were taken to monitor fetal and maternal wellbeing. Throughout the infusion period, blood was taken daily from the fetal artery for hormone analysis [9].

Between 128 and 131dGA, ewes and their fetuses were euthanised for tissue collection (200 mg/kg sodium pentobarbitone, iv.). A blood sample was taken from the umbilical artery before final euthanasia. Fetal brains were hemisected; fresh tissue samples (~10 mg) from the cerebrum (cerebral cortex, at the level of the ansate sulcus) and cerebellum (at the level of the horizontal fissure) were collected from the left hemisphere and placed in ice-cold buffer (miR05) [10]. The remaining portions of the left hemisphere were frozen in liquid nitrogen and stored at −80 °C for subsequent molecular analysis. The right hemisphere was immersion-fixed in 4% paraformaldehyde (PFA) for stereological analysis.

### 2.2. Cerebral Oxygen Consumption

Cerebral oxygen consumption was measured by high-resolution respirometry as previously described [10]. Fresh brain samples (~10 mg) were homogenised in miR05 and transferred into oxygraph chambers (Oxygraph 2k, Oroboros Instruments, Innsbruck, Austria). A substrate–inhibitor titration protocol was performed [11]. Nonphosphorylating leak respiration (CI_L_) was induced by adding the Complex I (CI)-linked substrates pyruvate (5 mM) and malate (2 mM). ADP (10 mM; saturating concentration) was added to stimulate OXPHOS. OXPHOS capacity of CI-linked substrates (CI_P_) was achieved by addition of glutamate (10 mM, saturating concentration). Maximal electron flux through Complex I and Complex II was achieved through addition of succinate (10 mM, CI&CII_P_). Subsequently, inhibition of CI by rotenone (0.5 µM) provided measurement of CII-linked OXPHOS capacity (CII_P_). Cytochrome *c* (10 µM) was added to check the integrity of the mitochondrial membranes with data excluded if respiration increased by >15%. Respiration rates were corrected for citrate synthase (CS) activity. Flux control ratios (FCRs) were calculated [10] as follows:

- The fraction of OXPHOS capacity dissipated in the leak state: C_L_/CI&CII_P_;
- OXPHOS coupling efficiency for CI-linked substrates, which represents the net OXPHOS capacity, corrected for leak respiration (CI_L_): 1-CI_L_/CI_P_;

To discern the fraction of OXPHOS capacity attributable to CI- and CII-linked respiration, FCRs were calculated as follows:

- CI_P_/CI&CII_P_ (Complex I Flux Control Ratio);
- CII_P_/CI&CII_P_ (Complex II Flux Control Ratio).

### 2.3. Biochemical Analyses

As several maturational processes during late gestation are regulated by hormones, including triiodothyronine (T_3_), which rises in a cortisol-dependent manner in the fetal circulation just before term [3], umbilical plasma triiodothyronine (T_3_) and thyroxine (T_4_) concentrations were measured by radioimmunoassay (MP Biomedicals, Loughborough, UK). Cortisol was measured by an ELISA kit (IBL International, Hamburg, Germany) [12]. The inter- and intra-assay coefficients of variations and minimum levels of detection were as previously reported [10].

Total protein (expressed as mg protein per gram wet weight) was measured in frozen brain samples using a bicinchoninic acid assay (Sigma-Aldrich, Gillingham, UK). Weighed cerebellar and cortical samples were freeze-dried for 24 h to measure water content.

CS is a putative marker of mitochondrial content [13,14]. Its activity was measured in 20 µg of the homogenised cerebellar and cortical protein samples by a spectrophotometric enzyme assay [15].

### 2.4. Western Blot Analyses

Total protein was extracted from frozen cerebellum and cortex samples (~50 mg) and separated by electrophoresis on a 12–14% polyacrylamide gel. Protein was transferred to a nitrocellulose membrane then stained with Ponceau-S to normalise for protein loading. Membranes were probed with an antibody cocktail to electron transfer system (ETS) complexes (OXPHOS antibody cocktail; Life Technologies, Carlsbad, CA, USA; 458099; 1:1000; RRID: AB_2533835) and adenine nucleotide translocase 1 (ANT1, Abcam, Cambridge, UK; ab102032; 1:1000; RRID:AB_10710263), followed by HRP-linked secondary antibody (GE Healthcare, Amersham, UK; NIF82; 1:5000). Protein bands were visualised using enhanced chemiluminescence then quantified using ImageJ (NIH).

### 2.5. Brain Histology and Stereology

The cerebellum was bisected at the midline of the vermis and the right side processed to paraffin wax and sagittally sectioned at 10 µm. The right cerebrum was cut coronally into 5 mm thick blocks, processed and embedded in paraffin. Sections (10 µm) were cut from three blocks (Level A, B, C, refer to Table 3) and sectioned as previously described [10]. Sections were stained with haematoxylin and eosin (H&E) to assess general morphology. As glucocorticoids play a critical role in myelination [16,17], immunostaining of myelin basic protein (MBP) was performed in both brain regions using immunohistochemistry (IHC) (MBP; Vector Laboratories, Newark, CA, USA; MAB386; 1:400; RRID:AB_94975; [10]). To assess the total area (H&E) and extent of myelination (MBP IHC), scanned sections of each brain region (NanoZoomer, Hamamatsu Photonics, Welwyn Garden City, UK) were converted to grayscale and the threshold was adjusted using ImageJ (NIH). The optical density (O.D.) of MBP staining was measured using ImageJ (NIH). Fields of view within the arbour vitae (cerebellum, *n* = 10/section), intragyral (cerebrum, *n* = 10/section) and periventricular white matter (cerebrum, *n* = 10/section) were sampled. All quantitative analyses were performed with the observer (E.J.C.) blind to the treatment groups.

### 2.6. Statistics

**Figure 1:**
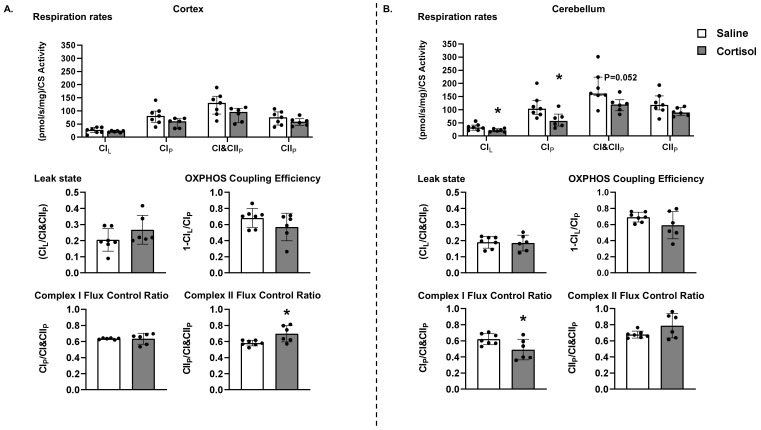
**Mitochondrial respiratory function in the cortex and cerebellum.** Data presented as median and interquartile range with individual data points (black dots) for respiratory rates relative to citrate synthase (CS) activity in the cortex (**A**) and cerebellum (**B**) for leak respiration (CI_L_), Complex I-linked respiration (CI_P_), CI&CII-linked respiration (CI&CII_P_) and CII_P_-linked respiration (CII_P_), leak state, oxidative phosphorylation (OXPHOS) coupling efficiency, the fraction of OXPHOS capacity attributable to CI and the fraction of OXPHOS capacity attributable to CII (CI and CII flux control ratios) from saline (open columns, *n* = 7) and cortisol-infused (filled columns, *n* = 6) fetuses at ~130 days of gestational age (dGA). * Significantly different from saline control, *p* < 0.05, *t*-test. One data point (Complex I Flux Control Ratio, control fetus) was detected as an outlier according to the ROUT method [18] and was removed from subsequent analysis.

All statistical analyses were performed using GraphPad Prism (version 9 for Windows, GraphPad Software, San Diego, CA, USA). A Shapiro–Wilk normality test was used to examine if data were normally distributed. Statistical outliers were detected using the ROUT method [18] using GraphPad Prism (version 9 for Windows, GraphPad Software, San Diego, CA, USA). One data point (Figure 1, noted in figure legend) was found to be an outlier according to this method and was removed from subsequent analysis. Values are presented as median with interquartile range (IQR, 25th–75th percentile). A *t*-test or Mann–Whitney nonparametric test were used to compare saline and cortisol-infused values, as appropriate. A repeated measures two-way ANOVA was used to compare cortisol concentration values at baseline and during the 5-day infusion period, followed by a Tukey’s post hoc test. Pearson’s correlation coefficient calculation was used to assess linear correlation between variables and log-transformed hormone concentrations. *p* Values of <0.05 were considered statistically significant.

## 3. Results

### 3.1. Cortisol Infusion Increases Cortisol and Plasma Triiodothyronine (T_3_) Concentrations and Citrate Synthase Activity

Relative to saline controls, the cortisol infusion resulted in elevated fetal cortisol concentrations within 24 h of beginning treatment, with concentrations remaining elevated thereafter at levels similar to those reported previously close to term [19,20]. Cortisol infusion also elevated plasma T_3_ concentrations at the end of infusion, and increased cerebellar, but not cortical, CS activity (Table 1). Cortisol infusion had no effect on plasma T_4_ concentration, morphometric measurements, or on water and protein content of either brain region (Table 1).

### 3.2. Cortisol Infusion Affects Cerebral Mitochondrial Respiratory Function in a Region-Specific Manner

To account for the regional differences in the mitochondrial content measured as CS activity, O_2_ consumption rates were normalised to CS activity in both brain regions to explore intrinsic changes in a respiratory capacity. In the cortex (Figure 1A), cortisol infusion had no effect on mitochondrial respiratory rates, leak state FCR, OXPHOS coupling efficiency, or on the contribution of CI to CI&CII_P_ OXPHOS capacity (CI FCR). However, the contribution of CII to the OXPHOS capacity (CII FCR) was elevated following cortisol infusion.

In the cerebellum (Figure 1B), CI_L_ and CI_P_ respiratory rates were reduced following cortisol infusion; CI&CII_P_ OXPHOS respiration also tended to be lower, but did not reach statistical significance (*p* = 0.052). The cortisol infusion had no effect on the leak state FCR, OXPHOS coupling efficiency, or CII FCR; however, the CI FCR was decreased in cortisol-infused fetuses.

When data from all fetuses were combined irrespective of treatment (Table 2), there was a significant positive correlation between cortical CI_P_ and CI&CII_P_ respiratory rates and the concentration of cortisol, but not T_3_. In the cerebellum, all respiratory rates, as well as CS activity, correlated with cortisol, but not T_3_ (Table 2).

### 3.3. Cortisol Infusion Does Not Alter the Protein Abundance of Mitochondrial Complexes, but Increases ANT1 in the Cerebellum

**Figure 2:**
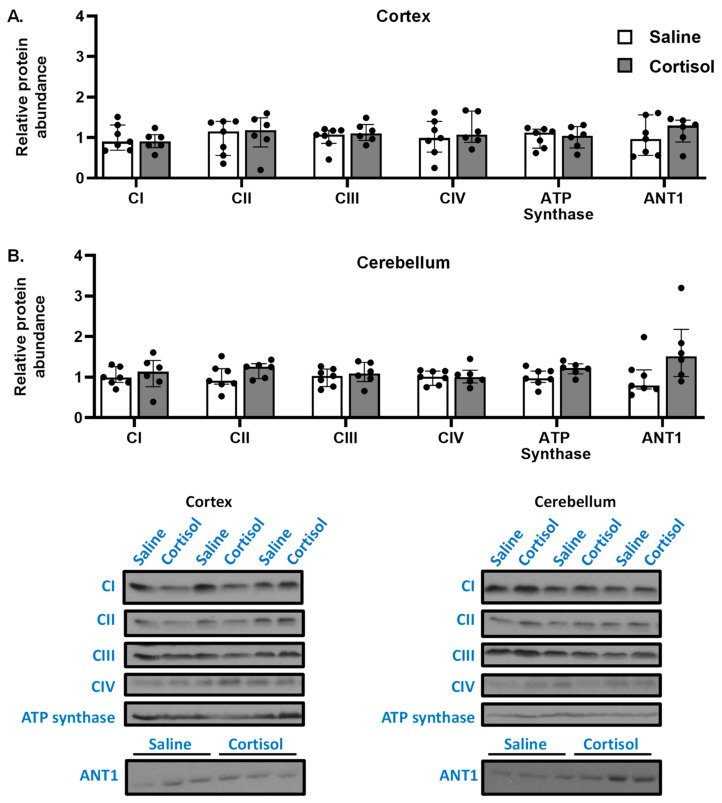
**Mitochondrial ETS complex and ANT1 abundance in the cerebellum and cortex.** Data presented as median and interquartile range with individual data points (black dots) for relative protein abundance in the cerebellum (**A**) and cortex (**B**) from saline (open columns, *n* = 7) and cortisol-infused (filled columns, *n* = 6) fetuses at ~130 days of gestational age (dGA).

Cortisol infusion had no effect on the abundances of Complexes I to IV or ATP synthase in either brain region (Figure 2). In the cerebellum (Figure 2B), but not the cortex (Figure 2A), the abundance of ANT1, a mitochondrial protein that facilitates the exchange of ADP and ATP across the mitochondrial inner membrane (Figure 2), tended to be increased following cortisol infusion, although this failed to reach significance.

### 3.4. Cortisol Infusion Increased Myelination in the Cerebrum

Cortisol infusion significantly increased the total area and proportion of myelin (%) in a subregion of the cerebrum relative to saline-infused fetuses (Table 3). The cerebellar morphology was unaffected by cortisol infusion (Table 3).

## 4. Discussion

The elevation of fetal cortisol concentrations within the physiological range near term altered cerebral mitochondrial content and OXPHOS capacity in a region-specific manner. These effects were accompanied by regional changes in cerebral myelination. Collectively, the findings highlighted the importance of cortisol in regulating the cerebral mitochondrial OXPHOS capacity of the fetal brain with implications for the transition from intra- to extrauterine life.

The findings showed that, despite increasing the cerebellar mitochondrial content, cortisol infusion reduced OXPHOS capacity per mitochondrial unit in the cerebellum. No changes in the abundance of ETS complexes or ATP synthase were observed. Glucocorticoids are known to influence mitochondrial biogenesis and dynamics in a range of adult tissues, including the brain [21,22,23,24,25]. The administration of potent synthetic glucocorticoids during rodent pregnancy has also been shown to increase the mitochondrial content in the fetal lung [26] and kidney [27], and alter the abundance of mitochondrial proteins in fetal tissues near term, such as the brain [28], kidney [27] and heart [29]. Recent studies have also shown that variations in the endogenous cortisol concentration affect CS activity in ovine fetal skeletal muscle [30].

The cortisol-induced increase in the cerebellar mitochondrial content in the current study may have been mediated by the upregulation of peroxisome proliferator-activated receptor gamma coactivator 1 alpha (PGC1α), the key regulator of mitochondrial biogenesis. Studies in rodents have reported that PGC1α increases in fetal heart and adipose tissue towards term and is glucocorticoid-sensitive [31,32]. Interestingly, ANT1 protein abundance in the cerebellum tended to increase following cortisol treatment. In adult rat liver, dexamethasone has been shown to increase the ANT1 content [33]. As well as functioning as a mitochondrial ADP–ATP exchanger, ANT1 induces mild mitochondrial uncoupling in adult liver and skeletal muscle [34,35,36]. Collectively, the current data suggest that, by stimulating mitochondrial biogenesis and ANT1, cortisol may increase the cerebellar capacity for ATP generation to meet the extra energy requirements associated with regulating movement and posture postnatally, while simultaneously reducing the risk of excessive reactive oxygen species (ROS) production during fluctuations in O_2_ availability during labour and delivery.

In contrast to the cerebellum, cortical mitochondrial content, OXPHOS capacity and ANT1 were not significantly altered following cortisol infusion. The differential effects of cortisol on the cortical and cerebellar OXPHOS capacity may reflect variations in glucocorticoid receptor expression or sensitivity of these brain regions to cortisol. These region-specific differences in OXPHOS capacity may also relate to the specific functions of the two regions and their relative importance in adapting to extrauterine life. The cerebellum is critical for controlling posture, locomotion and feeding, which are particularly important functions in precocial species such as sheep that must be able to stand, walk and suckle independently to survive after birth. The increase in the relative importance of CII, an integral component of the tricarboxylic acid (TCA) cycle, to OXPHOS capacity in both regions in response to cortisol infusion points towards an increased TCA cycle capacity in preparation for a greater oxidative metabolism in the more oxygen-rich extrauterine environment.

Since cortisol is known to be responsible for the normal prepartum rise in plasma T_3_ in fetal sheep by activating the tissue deiodinases that convert T_4_ to T_3_ peripherally [37], the regulatory effects of cortisol on mitochondrial function in the brain towards term may be partly mediated by T_3_ as occurs with other metabolic processes near term [38]. Recent studies in fetal sheep have demonstrated the importance of thyroid hormones in mitochondrial development towards term in both the brain [10] and skeletal muscle [15], although the current findings suggest that cortisol may be the predominant factor in regulating the prepartum maturation of cerebral mitochondrial OXPHOS capacity.

Elevating fetal cortisol concentrations near term also had regional effects on cerebral myelination, with an increase in the degree of myelination observed in the cerebrum, but not in the cerebellum. Glucocorticoids are known to play a role in normal brain development and myelination in the fetus. They regulate oligodendrocyte precursor differentiation to postmitotic oligodendrocytes, enhance the biosynthesis of myelin components and initiate myelin formation [16,17,39]. The process of myelination occurs in a caudal–rostral gradient [40]. The effect of cortisol infusion on myelination may, therefore, be dependent upon the specific brain region and developmental stage, in a pattern that follows the progression of the myelination ‘wave’.

Clinically, women at risk of preterm delivery are commonly treated with synthetic glucocorticoids (e.g., dexamethasone or betamethasone) to promote fetal lung maturation. Randomised trials have demonstrated that the administration of dexamethasone or betamethasone significantly reduces the incidences of respiratory distress syndrome (RDS), neonatal death, cerebral haemorrhage and necrotizing enterocolitis [41]. Similarly, trials of low-dose postnatal hydrocortisone supplementation in very preterm babies have suggested a benefit in terms of increased survival without bronchopulmonary dysplasia (BDP) [42] and improved neurodevelopmental outcomes [43]. The current results suggest that the benefits of glucocorticoid treatment on preterm outcomes may arise, in part, from the direct actions of the glucocorticoids on cerebral mitochondrial OXPHOS.

The central nervous system is a complex, integrated structure composed of diverse cell types. OXPHOS capacity was not examined in isolated cell types, which may be a limitation. The isolation of mitochondria requires large amounts of tissue and, therefore, may not be feasible when wanting to examine mitochondrial function in discrete brain regions during development. Using homogenates of brain tissue offers a high yield technique to assess OXPHOS capacity, with yields compa-rable or even giving superior results than with using isolated mitochondria [44], but with more direct physiologically relevance to mitochondrial function in situ in the whole animal [44,45]. To further elucidate the effect of cortisol on mitochondrial bioenergetics and dynamics, future studies should also assess the abundance and expression of mitochondrial regulatory proteins and genes within the brain.

In summary, the current study demonstrates the importance of cortisol in regulating cerebral mitochondrial capacity near term. These effects were region-specific, and involved changes in the mitochondrial content and respiratory function. While further studies are needed to identify the specific molecular pathways involved in the glucocorticoid regulation of cerebral mitochondrial function, the current findings have important implications for the health of infants born prematurely, or those overexposed to glucocorticoids by intrauterine stresses in compromised pregnancies or clinical treatment for threatened preterm labour.

## Figures and Tables

**Table 1:** **Fetal hormonal and biometric measurements.** Data are presented as median (interquartile range, IQR, 25th–75th percentile) from saline (*n* = 7) and cortisol-infused (*n* = 6) fetuses at ~130 days of gestational age (dGA). * Significantly different from saline control, *p* < 0.05, Mann–Whitney nonparametric test (for biparietal diameter) or *t*-test (remaining variables).

	Saline	Cortisol	*p* Value
	*n* = 4F, 3M	*n* = 4F, 2M	
	129.7 ± 0.5 dGA	130.3 ± 0.2 dGA	
**Hormone Concentrations**	**Median (IQR)**	**Median (IQR)**	
Plasma cortisol (ng/mL)	14.0 (8.5–16.6)	44.6 (36.6–51.1) *	<0.001
Plasma T_3_ (ng/mL)	0.39 (0.32–0.52)	0.77 (0.56–1.1) *	0.012
Plasma T_4_ (ng/mL)	137.2 (124.2–158.6)	142.7 (128.0–146.0)	0.911
**Morphometry**			
Body weight (kg)	3.1 (2.8–3.2)	2.7 (2.5–3.1)	0.276
Crown–rump length (cm)	44.0 (43.0–46.0)	44.0 (43.8–46.3)	0.657
Biparietal diameter (cm)	12.0 (11.0–12.0)	11.0 (10.5–11.6)	0.128
Brain weight (g)	42.8 (42.2–46.4)	40.4 (36.3–43.8)	0.096
Brain:body weight ratio (g:kg)	14.8 (13.4–15.2)	14.2 (11.8–16.6)	0.971
**Biochemical Composition**			
Cortex water content (%)	87.9 (87.6–88.7)	87.2 (86.0–87.4)	0.071
Cerebellum water content (%)	85.5 (84.2–85.7)	84.9 (84.1–85.8)	0.785
Protein cortex (mg/g)	36.3 (33.3–41.1)	37.6 (33.4–38.9)	0.582
Protein cerebellum (mg/g)	32.9 (30.8–45.0)	39.6 (30.5–49.1)	0.497
Cortex CS activity (μmol/min/mg protein)	0.228 (0.204–0.314)	0.237 (0.226–0.281)	0.966
Cerebellum CS activity (μmol/min/mg protein)	0.192 (0.161–0.217)	0.244 (0.227–0.281) *	0.015

**Table 2:** **Correlations of cortex and cerebellum mitochondrial respiration rates and citrate synthase activity with circulating plasma cortisol and T_3_.** Relationships between log10 plasma cortisol and T_3_ data and Complex I (CI)-linked leak respiration (CI_L_), CI (CI_P_), CI&II (CI&CII_P_) and CII (CII_P_)-linked OXPHOS capacity and citrate synthase (CS) activity. Data presented for all fetuses, saline and cortisol-infused (*n* = 12) at ~130 days of gestational age (dGA). *p* < 0.05, Pearson’s correlation coefficient.

Cortex	CI_L_	CI_P_	CI&II_P_	CII_P_	Citrate Synthase Activity
Log_10_ plasma cortisol (ng/mL)	*r* = −0.494	*r* = −0.629	*r* = −0.613	*r* = −0.491	*r* = 0.066
	*p* = 0.103	*p* = 0.028	*p* = 0.034	*p* = 0.105	*p* = 0.838
	*n* = 12	*n* = 12	*n* = 12	*n* = 12	*n* = 12
Log_10_ plasma T_3_ (ng/mL)	*r* = −0.153	*r* = −0.436	*r* = −0.519	*r* = −0.364	*r* = 0.245
	*p* = 0.635	*p* = 0.156	*p* = 0.083	*p* = 0.245	*p* = 0.442
	*n* = 12	*n* = 12	*n* = 12	*n* = 12	*n* = 12
**Cerebellum**	**CI_L_**	**CI_P_**	**CI + II_P_**	**CII**	**Citrate Synthase Activity**
Log_10_ plasma cortisol (ng/mL)	*r* = −0.664	*r* = −0.741	*r* = −0.718	*r* = −0.638	*r* = 0.692
	*p* = 0.024	*p* = 0.006	*p* = 0.009	*p* = 0.026	*p* = 0.013
	*n* = 12	*n* = 12	*n* = 12	*n* = 12	*n* = 12
Log_10_ plasma T_3_ (ng/mL)	*r* = −0.258	*r* = −0.347	*r* = −0.247	*r* = −0.075	*r* = 0.315
	*p* = 0.419	*p* = 0.270	*p* = 0.440	*p* = 0.818	*p* = 0.318
	*n* = 12	*n* = 12	*n* = 12	*n* = 12	*n* = 12

**Table 3:** **Morphology of the brain.** Data are presented as median (interquartile range, IQR, 25th–75th percentile) from saline (*n* = 7) and cortisol-infused (*n* = 6) fetuses at ~130 days of gestational age (dGA). * Significantly different from saline control, *p* < 0.05, Mann–Whitney nonparametric test (area of cerebrum, level C) or *t*-test (remaining variables). Below table, left: representative images showing the level (A, B and C) at which the brain regions were sectioned and stained with myelin basic protein (MBP); right: MBP-positive immunostaining of the cerebrum (level B) from a saline and a cortisol-infused fetus. Scale bar left, 1 cm; right, 2.5 mm.

Brain Region	Saline	Cortisol	*p* Value
	*n* = 4F, 3M	*n* = 4F, 2M	
**Cerebrum**			
**Level A**	**Median (IQR)**	**Median (IQR)**	
Area of cerebrum (mm^2^)	266.9 (253.6–311.4)	280.3 (252.9–304.9)	0.989
Area of myelin (mm^2^)	28.2 (25.8–30.2)	26.6 (21.2–30.1)	0.365
Myelin (%)	10.5 (8.7–11.6)	9.8 (7.8–11.0)	0.331
Myelin-periventricular (O.D.)	0.129 (0.108–0.136)	0.137 (0.120–0.148)	0.408
Myelin-intragyral (O.D.)	0.094 (0.082–0.127)	0.094 (0.087–0.107)	0.755
**Level B**			
Area of cerebrum (mm^2^)	402.8 (377.7–413.5)	404.3 (360.9–441.8)	0.888
Area of myelin (mm^2^)	80.4 (79.6–97.3)	114.3 (93.7–155.3) *	0.033
Myelin (%)	21.4 (19.8–23.5)	28.5 (25.9–34.3) *	0.003
Myelin-periventricular (O.D.)	0.166 (0.144–0.184)	0.187 (0.171–0.194)	0.135
Myelin-intragyral (O.D.)	0.106 (0.090–0.152)	0.116 (0.111–0.119)	0.904
**Level C**			
Area of cerebrum (mm^2^)	275.4 (226.3–323.9)	210.9 (209.7–282.3)	0.383
Area of myelin (mm^2^)	28.9 (19.7–41.0)	29.9 (28.0–43.8)	0.889
Myelin (%)	9.9 (8.0–18.1)	14.2 (13.3–15.5)	0.488
Myelin-periventricular (O.D.)	0.154 (0.112–0.186)	0.156 (0.147–0.165)	0.661
Myelin-intragyral (O.D.)	0.128 (0.067–0.151)	0.123 (0.107–0.144)	0.528
**Cerebellum**			
Area of cerebellum (mm^2^)	149.1 (146.8–170.1)	161.6 (125.2–194.1)	0.807
Area of myelin (mm^2^)	37.4 (34.1–41.1)	33.8 (30.1–45.1)	0.821
Myelin (%)	23.8 (20.1–29.5)	24.6 (17.6–27.6)	0.679
Myelin-arbour vitae, central (O.D.)	0.156 (0.141–0.159)	0.148 (0.130–0.157)	0.677
Myelin-arbour vitae, peripheral (O.D.)	0.189 (0.166–0.202)	0.181 (0.155–0.190)	0.773
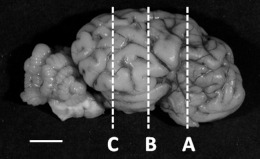	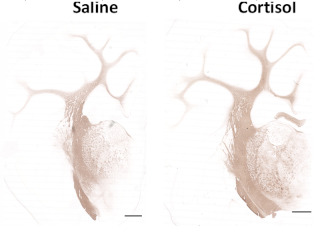		

## Data Availability

Some or all datasets generated during and/or analysed during the current study are not publicly available but are available from the corresponding author on reasonable request.
